# Supplementary material for: The Evolution and Characterization of the RNA Interference Pathways in Lophotrochozoa
Source: Genome Biol Evol. 2024 May 7;16(5):evae098. doi: 10.1093/gbe/evae098 (PMC11114477; doi:10.1093/gbe/evae098)
Supplement: evae098_Supplementary_Data [file evae098_supplementary_data.zip › Supplementary_materials.zip!/selected_miRNAs/NoGe_selected_miRNAs/mirdeep_pdf_files/NMRB01000754.1_Notospermus_geniculatus_strain_Ushimado_scaffold754,_whole_genome_shotgun_sequence_20348.pdf]

Provisional ID	:	NMRB01000754.1_Notospermus_geniculatus_strain_Ushimado_scaffold754,_whole_genome_shotgun_sequence_20348
Score total	:	46489.8
Score for star read(s)	:	3.9
Score for read counts	:	46480.9
Score for mfe	:	2.1
Score for randfold	:	0
Score for cons. seed	:	3
Total read count	:	91182
Mature read count	:	91084
Loop read count	:	0
Star read count	:	98

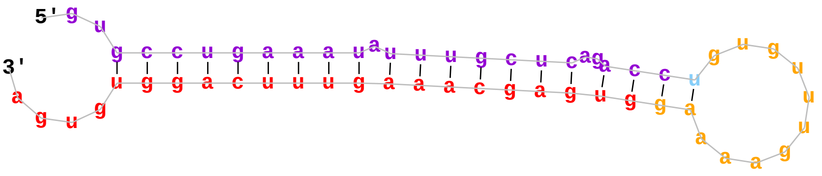

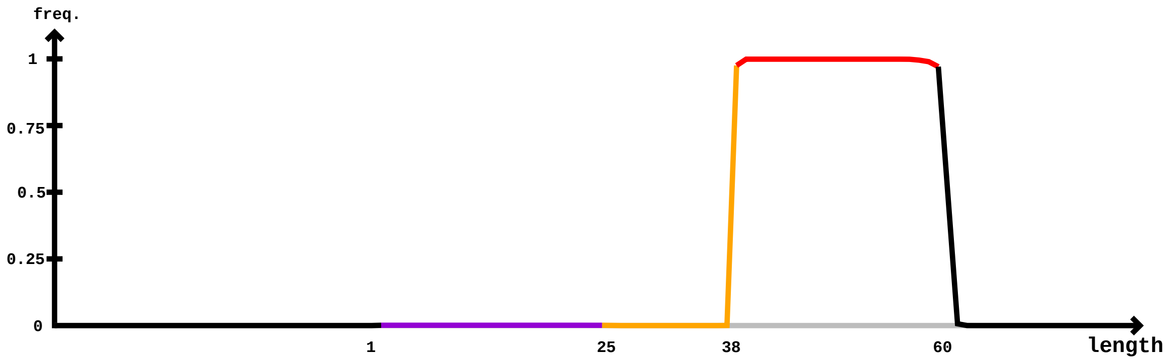

Star Mature

```
5'- aaaggauuaucaaauugaugucccugucaguugugccugaaauauuugcucagaccuguguuugaaaaggugagcaaaguuucaggugugacugauagguggcgugacagc -3'  obs
    aaaggauuaucaaauugaugucccugucaguugugccugaaauauuugcucagaccuguguuugaaaaggugagcaaaguuucaggugugacugauagguggcgugacagc      exp
    ...............(.((((((((((((((((((((((((((.(((((((..((((..........)))))))))))))))))))))))))))))))))).))))).)....  reads  mm  sample
    ................................gugccugaaauauuugcuca...........................................................  1  0  Mm2
    ................................gugccugaaauauuugcucaga.........................................................  1  0  Mm2
    ................................gugccugaaauauuugcucagacc.......................................................  5  0  Mm2
    ................................gugccugaaauauuugcucagaccu......................................................  6  0  Mm2
    ................................gugccugaaauauuugcucagaccugugu..................................................  1  0  Mm2
    ................................gugccugaaauauuugcucagaccugugG..................................................  1  1  Mm2
    ................................gugccugaaauauuugcucagaccuguguu.................................................  1  0  Mm2
    ....................................................................Cgugagcaaaguuucaggugugа....................  5  1  Mm2
    .....................................................................gugagcaaaguuucaggu........................  5  0  Mm2
    .....................................................................gugagcaaaguuucagguA.......................  1  1  Mm2
    .....................................................................gugagcaaaguuucaggug.......................  31  0  Mm2
    .....................................................................gugagcaaaguuucagCugu......................  1  1  Mm2
    .....................................................................gCgagcaaaguuucaggugu......................  2  1  Mm2
    .....................................................................gugagcaaaguuucaggCgu......................  2  1  Mm2
    .....................................................................gugagcaaaguuucaggugG......................  2  1  Mm2
    .....................................................................gugagcaaaguuucaggugu......................  49  0  Mm2
    .....................................................................gugagcaaaguuucagUugug.....................  1  1  Mm2
    .....................................................................gugagcaaaguuucaggAgug.....................  2  1  Mm2
    .....................................................................gugagcaaaguuucaggugug.....................  154  0  Mm2
    .....................................................................gugagcaaaguuucaggCgug.....................  3  1  Mm2
    .....................................................................gugagcaaaguuucaggGgug.....................  2  1  Mm2
    .....................................................................gugagcaaaguuucagguguU.....................  2  1  Mm2
    .....................................................................gugaUcaaaguuucaggugugа....................  1  1  Mm2
    .....................................................................gCgagcaaaguuucaggugugа....................  2  1  Mm2
    .....................................................................gugagcaaagGuucaggugugа....................  7  1  Mm2
    .....................................................................gugagcaGaguuucaggugugа....................  1  1  Mm2
    .....................................................................gugagcaaaguuuAaggugugа....................  2  1  Mm2
    .....................................................................gugagcaaaguuuUaggugugа....................  6  1  Mm2
    .....................................................................gugagcaUaguuucaggugugа....................  2  1  Mm2
    .....................................................................gugagNaaaguuucaggugugа....................  1  1  Mm2
    .....................................................................gugCgcaaaguuucaggugugа....................  2  1  Mm2
    .....................................................................gugagUaaaguuucaggugugа....................  2  1  Mm2
    .....................................................................gGgagcaaaguuucaggugugа....................  1  1  Mm2
```

```
                                Star                                Mature
aaaggauuaucaaauugaugucccugucaguugugccugaaauauuugcucagaccuguguuugaaaaggugagcaaaguuucaggugugacugauagguggcgugacagc
.....................................................................gugagcaaaguuucaggGguga....................    26     1    Mm2
.....................................................................gugagcaaagAuucaggugugа....................    14     1    Mm2
.....................................................................gugagcaaaguuucagCuguga....................    20     1    Mm2
.....................................................................gugaCcaaaguuucaggugugа....................    1      1    Mm2
.....................................................................gugagcUaaguuucaggugugа....................    2      1    Mm2
.....................................................................gugagcaaaguuucaggAguga....................    52     1    Mm2
.....................................................................gugagcGaaguuucaggugugа....................    1      1    Mm2
.....................................................................gugagcaaaguuucaggugCga....................    2      1    Mm2
.....................................................................gugagcaaaguuucaggugugC....................    12     1    Mm2
.....................................................................gugagcaaaguuucaggCguga....................    53     1    Mm2
.....................................................................gugagcaaaguuucagguAuga....................    1      1    Mm2
.....................................................................Uugagcaaaguuucaggugugа....................    3      1    Mm2
.....................................................................gugagcaaaguuucagAuguga....................    19     1    Mm2
.....................................................................gugagAaaaguuucaggugugа....................    1      1    Mm2
.....................................................................gugagcaaaguuucaCguguga....................    2      1    Mm2
.....................................................................gugagcaaaguuucaggugugG....................    1765   1    Mm2
.....................................................................gugagcaaaguuCcaggugugа....................    1      1    Mm2
.....................................................................gugagcaaaguuucGgguguga....................    3      1    Mm2
.....................................................................gugagcaaagCuucaggugugа....................    4      1    Mm2
.....................................................................gugagcaaaguuucagguUuga....................    1      1    Mm2
.....................................................................Nugagcaaaguuucaggugugа....................    4      1    Mm2
.....................................................................gugagcCaaguuucaggugugа....................    1      1    Mm2
.....................................................................gugagcaaCguuucaggugugа....................    2      1    Mm2
.....................................................................Augagcaaaguuucaggugugа....................    3      1    Mm2
.....................................................................gugagcaaaguuucaggugugU....................    183    1    Mm2
.....................................................................gugagcaCaguuucaggugugа....................    1      1    Mm2
.....................................................................gugagcaaaguuucaggugugа....................    7398   0    Mm2
.....................................................................gAgagcaaaguuucaggugugа....................    4      1    Mm2
.....................................................................gugagcaaaguuucagUuguga....................    9      1    Mm2
.....................................................................gugagcaaaguuucaggAgugac...................    1      1    Mm2
.....................................................................gugagcaaaguuucaggugugaU...................    296    1    Mm2
.....................................................................gugUgcaaaguuucaggugugac...................    1      1    Mm2
.....................................................................gugagcaaaguuucaggugugaG...................    6      1    Mm2
.....................................................................gugagcaaaguuucaggugugac...................    18     0    Mm2
.....................................................................gugagcaaaguuucaggugugaA...................    500    1    Mm2
.....................................................................gugagcaaaguuucaggugugaGu..................    5      1    Mm2
.....................................................................gugagcaaaguuucaggugugacu..................    5      0    Mm2
.....................................................................gugagcaaaguuucaggugugacC..................    6      1    Mm2
.....................................................................gugagcaaaguuucaggugugaAu..................    14     1    Mm2
.....................................................................gugagcaaaguuucaggugugaUu..................    37     1    Mm2
.....................................................................gugagcaaaguuucaggugugacA..................    2      1    Mm2
.....................................................................gugagcaaaguuucaggugugacuU.................    1      1    Mm2
......................................................................ugagcaaaguuucaggugug.....................    1      0    Mm2
......................................................................ugagcaaaguuucaggugugU....................    5      1    Mm2
......................................................................ugagcaaaguuucagguUuga....................    1      1    Mm2
......................................................................ugagcaaaguuucaggugugG....................    25     1    Mm2
......................................................................ugagcaaaguuucagguAuga....................    3      1    Mm2
......................................................................ugagcaaaguuucaggugugа....................    92     0    Mm2
......................................................................ugagcaaUguuucaggugugac...................    1      1    Mm2
......................................................................ugagcaaaguuucaggugugac...................    12     0    Mm2
......................................................................ugagcaaaguuucaggugugaU...................    16     1    Mm2
......................................................................ugagcaaaguuucaggugugaG...................    1      1    Mm2
......................................................................ugagcaaaguuucaggugugaA...................    8      1    Mm2
......................................................................ugagcaaaguuucaggugugaAu..................    1      1    Mm2
......................................................................ugagcaaUguuucaggugugacu..................    2      1    Mm2
......................................................................ugagcaaaguuucaggugugacu..................    1      0    Mm2
......................................................................ugagcaaaguuucaggugugaUu..................    1      1    Mm2

.....................................................................gugagcaaaguuucaggugu......................    15     0    Mm1
.....................................................................gAgagcaaaguuucaggugu......................    1      1    Mm1
.....................................................................gugagcaaagGuucaggugug.....................    1      1    Mm1
.....................................................................gugagcaaaguuucaggugug.....................    17     0    Mm1
.....................................................................gugagcUaaguuucaggugugа....................    1      1    Mm1
.....................................................................gAgagcaaaguuucaggugugа....................    1      1    Mm1
.....................................................................gugGgcaaaguuucaggugugа....................    1      1    Mm1
.....................................................................Uugagcaaaguuucaggugugа....................    4      1    Mm1
.....................................................................gugagcaaaguuuGaggugugа....................    1      1    Mm1
.....................................................................gugagcaaaguuucGgguguga....................    2      1    Mm1
.....................................................................gugagcaaaguuuUaggugugа....................    1      1    Mm1
.....................................................................guAagcaaaguuucaggugugа....................    2      1    Mm1
```

Star **Mature**

aaaggauuaucaaauugaugucccugucaguugugccugaaauauuugcucagaccuguguuugaaaaggugagcaaaguuucaggugugacugauagguggcgugacagc			
....gugagcaGaguuucagguguga....................	1	1	Mm1
....gugagcaaaguuucagguguga....................	3084	0	Mm1
....gugagcaaagCuucagguguga....................	3	1	Mm1
....gugagcaaaguuucagUuguga....................	4	1	Mm1
....Nugagcaaaguuucagguguga....................	2	1	Mm1
....gugUgcaaaguuucagguguga....................	1	1	Mm1
....gugagcaaaguuucaggAguga....................	15	1	Mm1
....gGgagcaaaguuucagguguga....................	1	1	Mm1
....gugagcaaaguuucagAuguga....................	7	1	Mm1
....gugagcaaaguuucCgguguga....................	1	1	Mm1
....gugaUcaaaguuucagguguga....................	2	1	Mm1
....gugagcaaaguuucaggCguga....................	12	1	Mm1
....gugagcaaaguuucaCguguga....................	1	1	Mm1
....gugagcaCaguuucagguguga....................	2	1	Mm1
....gugagcaaagGuucagguguga....................	5	1	Mm1
....gugagcaUaguuucagguguga....................	1	1	Mm1
....gugagcaaaguuucaggGguga....................	12	1	Mm1
....gugagcaaagAuucagguguga....................	3	1	Mm1
....gugagcaaaguuucagguUuga....................	2	1	Mm1
....gugagcaaaguuucaggugugG....................	130	1	Mm1
....gugagcaaaguuucaggugugU....................	97	1	Mm1
....gugagcaaaguuucaUguguga....................	1	1	Mm1
....gugagcaaaguuucagguguUa....................	1	1	Mm1
....gugagcaaaguuucagCuguga....................	8	1	Mm1
....gugagAaaaguuucagguguga....................	1	1	Mm1
....gugagcaaaguuucaggugugaG...................	6	1	Mm1
....gugagcaaaguuucaggugugaU...................	77	1	Mm1
....gugagcaaaguuucaggugugaA...................	38	1	Mm1
....gugagcaaaguuucaggugugaAu..................	23	1	Mm1
....ugagcaaaguuucagguguga....................	22	0	Mm1
....ugagcaaaguuucagguAuga....................	1	1	Mm1
....ugagcaaaguuucaggugugG....................	2	1	Mm1
................................gugccugaaauauuugcucagacc...	16	0	Fm2
....Cgugagcaaaguuucagguguga....................	11	1	Fm2
....gugagcaaaguuucaggu........................	2	0	Fm2
....gugagcaaaguuucaggug.......................	28	0	Fm2
....gugagcaaaguuucagguU.......................	3	1	Fm2
....gugagcaaaguuucaggAg.......................	1	1	Fm2
....gugagcaaaguuucagguA.......................	5	1	Fm2
....gugagcaaaguuucaggugG......................	5	1	Fm2
....gugagcaaaguuucaggugu......................	63	0	Fm2
....gugagcaaaguuucaggAgu......................	1	1	Fm2
....gugagcaaaguuucaggGgug.....................	1	1	Fm2
....gugagcaaaguuucaggugug.....................	287	0	Fm2
....gugagcaaaguuucaggAgug.....................	1	1	Fm2
....gugagcaaaguuucaggCgug.....................	1	1	Fm2
....gugagcaaagAuucaggugug.....................	3	1	Fm2
....gugGgcaaaguuucagguguga....................	4	1	Fm2
....gugagcaaaguuucaggAguga....................	43	1	Fm2
....gugagNaaaguuucagguguga....................	4	1	Fm2
....gugagcaaaguuucagguguAa....................	1	1	Fm2
....gugUgcaaaguuucagguguga....................	2	1	Fm2
....gugagcaaGguuucagguguga....................	1	1	Fm2
....gugagcaCaguuucagguguga....................	2	1	Fm2
....gugagcaaagGuucagguguga....................	4	1	Fm2
....gugagcaaaguuucagCuguga....................	21	1	Fm2
....gugagcaaagAuucagguguga....................	15	1	Fm2
....gugagcaaCguuucagguguga....................	2	1	Fm2
....gugagcaaaguuucaUguguga....................	1	1	Fm2
....gugaAcaaaguuucagguguga....................	1	1	Fm2
....gAgagcaaaguuucagguguga....................	1	1	Fm2
....gugagcaaaguuucagguguga....................	7706	0	Fm2
....gugagcaaagCuucagguguga....................	5	1	Fm2
....gugagcaGaguuucagguguga....................	3	1	Fm2
....gugagcaaaguuucaggGguga....................	40	1	Fm2
....gugagcaaaguuucagUuguga....................	11	1	Fm2
....guUagcaaaguuucagguguga....................	3	1	Fm2
....gugagcGaaguuucagguguga....................	1	1	Fm2
....gugagcaaaguuucaggugCga....................	2	1	Fm2

Star **Mature**

```
aaaggauuaucaaauugaugucccugucaguugugccugaaauauuugcucagaccuguguuugaaaaggugagcaaaguuucagguguga cugauagguggcgugacagc
.....................................................................gugagcaaaguuucagAuguga....................   16     1   Fm2
.....................................................................gugCgcaaaguuucagguguga....................   2      1   Fm2
.....................................................................Uugagcaaaguuucagguguga....................   7      1   Fm2
.....................................................................gugagcaaaguuGcagguguga....................   1      1   Fm2
.....................................................................gugagcaaaguuucaggugugU....................   200    1   Fm2
.....................................................................gugagcaaaguuucGgguguga....................   6      1   Fm2
.....................................................................Nugagcaaaguuucagguguga....................   2      1   Fm2
.....................................................................guCagcaaaguuucagguguga....................   3      1   Fm2
.....................................................................Cugagcaaaguuucagguguga....................   1      1   Fm2
.....................................................................gugagAaaaguuucagguguga....................   1      1   Fm2
.....................................................................gugagcaaaguCucagguguga....................   1      1   Fm2
.....................................................................gugagcaaaguuucUgguguga....................   1      1   Fm2
.....................................................................guAagcaaaguuucagguguga....................   5      1   Fm2
.....................................................................gugagcaaaguuucaggugugC....................   11     1   Fm2
.....................................................................gugagcaaaguuucagguguUa....................   7      1   Fm2
.....................................................................gugagcaaaguuucagguAuga....................   1      1   Fm2
.....................................................................gugagcaaaguuucaggugugG....................   2130   1   Fm2
.....................................................................gugagcaUaguuucagguguga....................   2      1   Fm2
.....................................................................Augagcaaaguuucagguguga....................   1      1   Fm2
.....................................................................gugagcaaaguuCcagguguga....................   2      1   Fm2
.....................................................................gugagcaaaguuucaggCguga....................   41     1   Fm2
.....................................................................gugagcaaUguuucaggugugac...................   8      1   Fm2
.....................................................................gugagcaaaguuucaggugugac...................   20     0   Fm2
.....................................................................gugagcaaaguuucaggugugaU...................   360    1   Fm2
.....................................................................gugagcaaaguuucaggugugaG...................   11     1   Fm2
.....................................................................gugagcaaaguuucaggGgugac...................   1      1   Fm2
.....................................................................gugagcaaaguuucaggugugaA...................   539    1   Fm2
.....................................................................gugagcaaaguuucaggCgugacu..................   1      1   Fm2
.....................................................................gugagcaaaguuucaggugugacC..................   17     1   Fm2
.....................................................................gugagcaaaguuucaggugugacG..................   1      1   Fm2
.....................................................................gugagcaaaguuucaggugugaAu..................   30     1   Fm2
.....................................................................gugagcaaaguuucaggugugaGu..................   22     1   Fm2
.....................................................................gugagcaaaguuucaggugugaUu..................   61     1   Fm2
.....................................................................gugagcaaaguuucaggugugacu..................   14     0   Fm2
......................................................................ugagcaaagCuucagguguga....................   1      1   Fm2
......................................................................ugagcaaaguuucaggugugG....................   35     1   Fm2
......................................................................ugagcaaaguuucaggugugU....................   7      1   Fm2
......................................................................ugagcGaaguuucagguguga....................   1      1   Fm2
......................................................................ugagcaaaguuucagguguga....................   116    0   Fm2
......................................................................ugagcaaUguuucaggugugac...................   7      1   Fm2
......................................................................ugagcaaaguuucaggugugac...................   22     0   Fm2
......................................................................ugagcaaaguuucaggugugaU...................   20     1   Fm2
......................................................................ugagcaaaguAucaggugugacu..................   1      1   Fm2
......................................................................ugagcaaaguuucaggugugaUu..................   17     1   Fm2
......................................................................ugagcaaaguuucaggugugacu..................   27     0   Fm2

................................gugccugaaauauuugcucaga.........................................................   2      0   Fm1
................................gugccugaaauauuugcucagacc.......................................................   27     0   Fm1
................................gugccugaaauauuugcucagaccu......................................................   29     0   Fm1
................................gugccugaaauauuugcucagaccG......................................................   3      1   Fm1
...................................................................Gggugagcaaaguuucagguguga....................   3      1   Fm1
....................................................................Cgugagcaaaguuucagguguga....................   3      1   Fm1
.....................................................................gugagcaaaguuucaggu........................   17     0   Fm1
.....................................................................Augagcaaaguuucaggug.......................   1      1   Fm1
.....................................................................Nugagcaaaguuucaggug.......................   1      1   Fm1
.....................................................................gugagcaaaguuucaggCg.......................   4      1   Fm1
.....................................................................gugagcaaaguuucagguU.......................   4      1   Fm1
.....................................................................gugagcaaaguuucaggAg.......................   1      1   Fm1
.....................................................................gugagcaaaguuucaggug.......................   156    0   Fm1
.....................................................................gugagcaaaguuucagguA.......................   7      1   Fm1
.....................................................................gugagcaaaguuucaggGgu......................   2      1   Fm1
.....................................................................gugagcaaaguuucagAugu......................   1      1   Fm1
.....................................................................gugagcaaaguuucaggugG......................   19     1   Fm1
.....................................................................gugagcaaaguuucaggugA......................   3      1   Fm1
.....................................................................gugagcaaaguuucaggCgu......................   3      1   Fm1
.....................................................................guUagcaaaguuucaggugu......................   1      1   Fm1
.....................................................................gugagcaaaguuucaggugu......................   217    0   Fm1
.....................................................................gugagcUaaguuucaggugug.....................   1      1   Fm1
.....................................................................guAagcaaaguuucaggugug.....................   1      1   Fm1
.....................................................................gugagcaaaguuucagAugug.....................   1      1   Fm1
```

Star Mature

```
aaaggauuaucaaauugaugucccugucaguugugccugaaauauuugcucagaccuguguuugaaaaggugagcaaaguuucaggugugacugauagguggcgugacagc
.....................................................................gugagcaGaguuucaggugug.....................  1      1   Fm1
.....................................................................gugagcaaagGuucaggugug.....................  1      1   Fm1
.....................................................................gCgagcaaaguuucaggugug.....................  1      1   Fm1
.....................................................................gugagcaaaguuuGaggugug.....................  1      1   Fm1
.....................................................................gugGgcaaaguuucaggugug.....................  1      1   Fm1
.....................................................................gugagcaaaguuucaggGgug.....................  4      1   Fm1
.....................................................................Uugagcaaaguuucaggugug.....................  1      1   Fm1
.....................................................................gugagcaaaguuucaggAgug.....................  3      1   Fm1
.....................................................................gugCgcaaaguuucaggugug.....................  1      1   Fm1
.....................................................................gugagcaaaguuuAaggugug.....................  1      1   Fm1
.....................................................................gugagcaaaguuucaggCgug.....................  4      1   Fm1
.....................................................................gugagcaaaguuucGggugug.....................  1      1   Fm1
.....................................................................gugagcaaaguuucagguguU.....................  10     1   Fm1
.....................................................................gugagcaaaguuucaggugGg.....................  1      1   Fm1
.....................................................................gugagcaaagAuucaggugug.....................  3      1   Fm1
.....................................................................gugagcaaaguuucaggugug.....................  512    0   Fm1
.....................................................................gugagcaaaguuucagguguA.....................  4      1   Fm1
.....................................................................gugagNaaaguuucaggugugа....................  1      1   Fm1
.....................................................................gugagcaaaguuucagguguUa....................  2      1   Fm1
.....................................................................gugagcaGaguuucaggugugа....................  1      1   Fm1
.....................................................................gugagAaaaguuucaggugugа....................  1      1   Fm1
.....................................................................gugagcaaaguuucagguguCa....................  1      1   Fm1
.....................................................................gugagcaaaCuuucaggugugа....................  1      1   Fm1
.....................................................................gugagcaaaguuucaggugAga....................  2      1   Fm1
.....................................................................gugagcaaaguCucaggugugа....................  1      1   Fm1
.....................................................................gugaUcaaaguuucaggugugа....................  1      1   Fm1
.....................................................................Augagcaaaguuucaggugugа....................  5      1   Fm1
.....................................................................gugagcaaaguuucagCuguga....................  36     1   Fm1
.....................................................................guUagcaaaguuucaggugugа....................  5      1   Fm1
.....................................................................gugagcaaCguuucaggugugа....................  2      1   Fm1
.....................................................................gugagGaaaguuucaggugugа....................  1      1   Fm1
.....................................................................gugaCcaaaguuucaggugugа....................  1      1   Fm1
.....................................................................Uugagcaaaguuucaggugugа....................  12     1   Fm1
.....................................................................gugagcaaaguuucaggAguga....................  72     1   Fm1
.....................................................................gugaAcaaaguuucaggugugа....................  3      1   Fm1
.....................................................................gugagcaaaguuucaggugugа....................  12695  0   Fm1
.....................................................................gugUgcaaaguuucaggugugа....................  2      1   Fm1
.....................................................................guCagcaaaguuucaggugugа....................  1      1   Fm1
.....................................................................gugagcaaaguuuUaggugugа....................  10     1   Fm1
.....................................................................gugCgcaaaguuucaggugugа....................  4      1   Fm1
.....................................................................gCgagcaaaguuucaggugugа....................  4      1   Fm1
.....................................................................gugagcaaaguuucaggGguga....................  65     1   Fm1
.....................................................................guAagcaaaguuucaggugugа....................  3      1   Fm1
.....................................................................gugagcaaagGuucaggugugа....................  13     1   Fm1
.....................................................................gugagcaaaguuucaggugugG....................  5013   1   Fm1
.....................................................................gugagcaaaguuucaggCguga....................  83     1   Fm1
.....................................................................gugagcaaaguuucagAuguga....................  33     1   Fm1
.....................................................................gAgagcaaaguuucaggugugа....................  7      1   Fm1
.....................................................................gugagcaaaguuucaggugugC....................  62     1   Fm1
.....................................................................gugagcaaagCuucaggugugа....................  13     1   Fm1
.....................................................................gugagcaaaguAucaggugugа....................  1      1   Fm1
.....................................................................gugagcaaaguuGcaggugugа....................  1      1   Fm1
.....................................................................gugagcaaaguuucagUuguga....................  21     1   Fm1
.....................................................................gugagcaCaguuucaggugugа....................  1      1   Fm1
.....................................................................gugagcGaaguuucaggugugа....................  4      1   Fm1
.....................................................................gugagcaaGguuucaggugugа....................  1      1   Fm1
.....................................................................gugagcaUaguuucaggugugа....................  1      1   Fm1
.....................................................................gugagcaaaguuucaggugCga....................  2      1   Fm1
.....................................................................gugagUaaaguuucaggugugа....................  2      1   Fm1
.....................................................................gugagcaaaguuucagguAuga....................  1      1   Fm1
.....................................................................gugagcaaaguuucGggugugа....................  7      1   Fm1
.....................................................................gugagcaaaguuucaggugugU....................  478    1   Fm1
.....................................................................gGgagcaaaguuucaggugugа....................  2      1   Fm1
.....................................................................gugagcaaaguuuAaggugugа....................  4      1   Fm1
.....................................................................Nugagcaaaguuucaggugugа....................  5      1   Fm1
.....................................................................gugagcaaagAuucaggugugа....................  23     1   Fm1
.....................................................................gugagcNaaguuucaggugugа....................  1      1   Fm1
.....................................................................gugGgcaaaguuucaggugugа....................  3      1   Fm1
.....................................................................gugagcaaaUuucaggugugа....................  1      1   Fm1
.....................................................................gugagcUaaguuucaggugugа....................  3      1   Fm1
```

```
                                Star                                 Mature
aaaggauuaucaaauugaugucccugucaguugugccugaaauauuugcucagaccuguguuugaaaaggugagcaaaguuucaggugugacugauagguggcgugacagc
.....................................................................gugagcaaaguuCcagguguga....................  1    1  Fm1
.....................................................................gugagcaaaguuucUgguguga....................  1    1  Fm1
.....................................................................gugagcaaaguuAcagguguga....................  1    1  Fm1
.....................................................................gugagcaaUguuucaggugugac...................  3    1  Fm1
.....................................................................gugagcaaaguuucaggugugCc...................  1    1  Fm1
.....................................................................gugagcaaaguuucaggugugaA...................  847  1  Fm1
.....................................................................gugagcaaaguuucaggugugac...................  78   0  Fm1
.....................................................................guAagcaaaguuucaggugugac...................  1    1  Fm1
.....................................................................gugagcaaaguuucaggugugaG...................  29   1  Fm1
.....................................................................gugagcaaaguuucaggugugaU...................  617  1  Fm1
.....................................................................gugagcaaaguuucaggugugacC..................  12   1  Fm1
.....................................................................gugagcaaaguuucaggugugaAu..................  46   1  Fm1
.....................................................................gugagcaaaguuucaggugugaUu..................  85   1  Fm1
.....................................................................gugagcaaaguuucaggugugaGu..................  11   1  Fm1
.....................................................................gugagcaaaguuucaggugugacu..................  9    0  Fm1
.....................................................................gugagcaaaguuucaggugugacCg.................  1    1  Fm1
......................................................................ugagcaaaguGucagguguga....................  1    1  Fm1
......................................................................ugagcaaaguuucUgguguga....................  1    1  Fm1
......................................................................ugagcaGaguuucagguguga....................  1    1  Fm1
......................................................................ugagcaaaguuucaggugugU....................  13   1  Fm1
......................................................................ugagcaaaguuucaggAguga....................  2    1  Fm1
......................................................................ugagcaaaguuucagguguga....................  141  0  Fm1
......................................................................ugagcaaaguuucaggugugG....................  51   1  Fm1
......................................................................ugagcaaaguuucaggugugac...................  24   0  Fm1
......................................................................ugagcaaUguuucaggugugac...................  7    1  Fm1
......................................................................ugagcaaaguuucaggugugaA...................  14   1  Fm1
......................................................................ugagcaaaguuucaggugugaU...................  17   1  Fm1
......................................................................ugagcaaaguuucaggugugaUu..................  4    1  Fm1
......................................................................ugagcaaaguuucaggugugacu..................  3    0  Fm1
......................................................................ugagcaaaguuucaggugugacuU.................  5    1  Fm1
.......................................................................gagcaaaguuucaggugug.....................  3    0  Fm1

................................gugccugaaauauuugcucagaccu......................................................  2    0  Mm3
....................................................................Cgugagcaaaguuucagguguga....................  1    1  Mm3
.....................................................................gugagcaaaguuucaggu........................  1    0  Mm3
.....................................................................gugagcaaaguuucaggug.......................  7    0  Mm3
.....................................................................gugagcaaaguuucaggGgu......................  1    1  Mm3
.....................................................................gugagcaaaguuucaggCgu......................  1    1  Mm3
.....................................................................gugagcaaaguuucaggugG......................  1    1  Mm3
.....................................................................gugagcaaaguuucaggugA......................  1    1  Mm3
.....................................................................gugagcaaaguuucaggugu......................  45   0  Mm3
.....................................................................gugagUaaaguuucaggugug.....................  1    1  Mm3
.....................................................................gugagcaaagAuucaggugug.....................  1    1  Mm3
.....................................................................gugagcaaaguuucaggugug.....................  100  0  Mm3
.....................................................................gugagcaaaguuucaggAgug.....................  1    1  Mm3
.....................................................................Nugagcaaaguuucaggugug.....................  1    1  Mm3
.....................................................................gCgagcaaaguuucagguguga....................  1    1  Mm3
.....................................................................gugagcaaaguuucagUuguga....................  4    1  Mm3
.....................................................................gugagcaaaguuucagCuguga....................  15   1  Mm3
.....................................................................gugagcaaaguuucaggugugU....................  153  1  Mm3
.....................................................................gGgagcaaaguuucagguguga....................  2    1  Mm3
.....................................................................Nugagcaaaguuucagguguga....................  1    1  Mm3
.....................................................................gugagcaaaguuCcagguguga....................  1    1  Mm3
.....................................................................gugUgcaaaguuucagguguga....................  2    1  Mm3
.....................................................................gugagcaaaguuucaggugugG....................  709  1  Mm3
.....................................................................gugagcUaaguuucagguguga....................  1    1  Mm3
.....................................................................gugagcaaaguuucaggugugC....................  4    1  Mm3
.....................................................................gugCgcaaaguuucagguguga....................  2    1  Mm3
.....................................................................gugagcaaaguuucGgguguga....................  2    1  Mm3
.....................................................................gugagcaaaguuucagguUuga....................  2    1  Mm3
.....................................................................gugagNaaaguuucagguguga....................  1    1  Mm3
.....................................................................gugagcaaaguuucUgguguga....................  1    1  Mm3
.....................................................................gugGgcaaaguuucagguguga....................  2    1  Mm3
.....................................................................gugagcaaagCuucagguguga....................  2    1  Mm3
.....................................................................gugagcaaaguuucaggCguga....................  23   1  Mm3
.....................................................................gugagcaaaguuUagguguga....................  2    1  Mm3
.....................................................................gugagcaaaguuucaggugAga....................  1    1  Mm3
.....................................................................gugagcaaCguuucagguguga....................  1    1  Mm3
.....................................................................gugagcaaaguuucagAuguga....................  13   1  Mm3
.....................................................................gugagcaCaguuucagguguga....................  3    1  Mm3
```

```
                               Star                               Mature
aaaggauuaucaaauugaugucccugucaguugugccugaaauauuugcucagaccuguguuugaaaaggugagcaaaguuucaggugugacugauagguggcgugacagc
.....................................................................gugaUcaaaguuucagguguga....................  1      1   Mm3
.....................................................................gugagcaaaguCucagguguga....................  1      1   Mm3
.....................................................................gugagcGaaguuucagguguga....................  1      1   Mm3
.....................................................................gugagcaaaguuucaggugugUa....................  4      1   Mm3
.....................................................................gugagcaaagGuucagguguga....................  9      1   Mm3
.....................................................................gAgagcaaaguuucagguguga....................  3      1   Mm3
.....................................................................gugagcaaaguuucaUguguga....................  1      1   Mm3
.....................................................................gugagcaaaguuucaggAguga....................  27     1   Mm3
.....................................................................guAagcaaaguuucagguguga....................  1      1   Mm3
.....................................................................gugagcaaaguuucaggGguga....................  14     1   Mm3
.....................................................................Uugagcaaaguuucagguguga....................  2      1   Mm3
.....................................................................gugagcaaaguuucagguguga....................  5121   0   Mm3
.....................................................................gugagcaaagAuucagguguga....................  8      1   Mm3
.....................................................................guUagcaaaguuucagguguga....................  1      1   Mm3
.....................................................................gugagcaaaguuucaggugugac...................  15     0   Mm3
.....................................................................gugagcaaaguuucaggugugaU...................  178    1   Mm3
.....................................................................gugagcaaaguuucaggugugaG...................  4      1   Mm3
.....................................................................gugagcaaaguuucaggugugaA...................  271    1   Mm3
.....................................................................gugagcaaaguuucaggugugaAu..................  17     1   Mm3
.....................................................................gugagcaaaguuucaggugugaUu..................  29     1   Mm3
.....................................................................gugagcaaaguuucaggugugacu..................  2      0   Mm3
.....................................................................gugagcaaaguuucaggugugaGu..................  2      1   Mm3
......................................................................ugagcaaaguuucaggugug.....................  2      0   Mm3
......................................................................ugagcaaaguuucagguAuga....................  2      1   Mm3
......................................................................ugagcaaaguuucagguUuga....................  1      1   Mm3
......................................................................ugagcaaaguGucagguguga....................  1      1   Mm3
......................................................................ugagcaaaguuucagguguga....................  75     0   Mm3
......................................................................ugagcaaaguuucaggugugU....................  2      1   Mm3
......................................................................ugagcaaaguuucaggugugG....................  12     1   Mm3
......................................................................ugagcaaUguuucaggugugac...................  1      1   Mm3
......................................................................ugagcaaaguuucaggugugaU...................  8      1   Mm3
......................................................................ugagcaaaguuucaggugugac...................  4      0   Mm3
......................................................................ugagcaaaguuucaggugugaA...................  7      1   Mm3
......................................................................ugagcaaaguuucaggugugacu..................  1      0   Mm3
......................................................................ugagcaaaguuucaggugugacA..................  1      1   Mm3

................................gugccugaaauauuugcucagacc.......................................................  3      0   Fm3
....................................................................Cgugagcaaaguuucagguguga....................  12     1   Fm3
.....................................................................gugagcaaaguuucaggu........................  1      0   Fm3
.....................................................................gugagcaaaguuucaggug.......................  57     0   Fm3
.....................................................................gugagcaaaguuucagguA.......................  1      1   Fm3
.....................................................................gugagcaaaguuucaggugu......................  84     0   Fm3
.....................................................................gugagcaaaguuucaggGgu......................  1      1   Fm3
.....................................................................gugagcaaaguuucaggAgu......................  2      1   Fm3
.....................................................................gugagcaaaguuucaggugA......................  3      1   Fm3
.....................................................................gugagcGaaguuucaggugug.....................  1      1   Fm3
.....................................................................gugagcaaaguuucaggGgug.....................  3      1   Fm3
.....................................................................gugagcaaaguuucagUugug.....................  2      1   Fm3
.....................................................................gugagcaaaguuucagCugug.....................  2      1   Fm3
.....................................................................gugagcaaagAuucaggugug.....................  1      1   Fm3
.....................................................................gugagcaaaguuucaggCgug.....................  5      1   Fm3
.....................................................................gugagcUaaguuucaggugug.....................  1      1   Fm3
.....................................................................gugagNaaaguuucaggugug.....................  1      1   Fm3
.....................................................................gugagcaaaguuucaggAgug.....................  2      1   Fm3
.....................................................................gugagcaaaguuuAaggugug.....................  1      1   Fm3
.....................................................................gugagcaaGguuucaggugug.....................  1      1   Fm3
.....................................................................gugagcaaaguuucaggugug.....................  549    0   Fm3
.....................................................................gugagcaaaguuucagguguCa....................  2      1   Fm3
.....................................................................gugagAaaaguuucagguguga....................  2      1   Fm3
.....................................................................gugaCcaaaguuucagguguga....................  5      1   Fm3
.....................................................................gugGgcaaaguuucagguguga....................  4      1   Fm3
.....................................................................gugagcaaaguuucaAguguga....................  1      1   Fm3
.....................................................................gugagcaGaguuucagguguga....................  2      1   Fm3
.....................................................................gugagcaaaguuUagguguga....................  34     1   Fm3
.....................................................................gugagcaaaguuucGgguguga....................  3      1   Fm3
.....................................................................Cugagcaaaguuucagguguga....................  1      1   Fm3
.....................................................................gugCgcaaaguuucagguguga....................  10     1   Fm3
.....................................................................gugagcaaaguuucaggGguga....................  127    1   Fm3
.....................................................................gugagcaaaguuucagguguga....................  29819  0   Fm3
.....................................................................gugagcaaaguuAcagguguga....................  1      1   Fm3
```

Star Mature

```
aaaggauuaucaaauugaugucccugucaguugugccugaaauauuugcucagaccuguguuugaaaaggugagcaaaguuucaggugugacugauaggugcgugacagc
.....................................................................guAagcaaaguuucaggugugа................... 10 1 Fm3
.....................................................................gugagcaaaguuucaggugugC................... 14 1 Fm3
.....................................................................gGgagcaaaguuucaggugugа................... 4 1 Fm3
.....................................................................gugagcaaaguuucaUgugugа................... 12 1 Fm3
.....................................................................gugagcaaaguuuAaggugugа................... 8 1 Fm3
.....................................................................gugagcaaagGuucaggugugа................... 52 1 Fm3
.....................................................................gugagNaaaguuucaggugugа................... 5 1 Fm3
.....................................................................gugagUaaaguuucaggugugа................... 3 1 Fm3
.....................................................................gugagcaaaguuucaggugGga................... 4 1 Fm3
.....................................................................gugagcaaaguuucaggugAga................... 7 1 Fm3
.....................................................................gugagcaaaguuucagguUuga................... 2 1 Fm3
.....................................................................gugagcaaaUuuucaggugugа................... 3 1 Fm3
.....................................................................gugagcaaaguuucagguAuga................... 6 1 Fm3
.....................................................................gugagcaaaguuucaggugugU................... 1002 1 Fm3
.....................................................................Uugagcaaaguuucaggugugа................... 12 1 Fm3
.....................................................................gugagcaaaguuucaCgugugа................... 1 1 Fm3
.....................................................................gugagcaUaguuucaggugugа................... 14 1 Fm3
.....................................................................gugagcaaCguuucaggugugа................... 8 1 Fm3
.....................................................................gugagcaaaguuucagguguAa................... 6 1 Fm3
.....................................................................guUagcaaaguuucaggugugа................... 4 1 Fm3
.....................................................................gugagcaaaAuuucaggugugа................... 1 1 Fm3
.....................................................................gugagcaaaguuCcaggugugа................... 2 1 Fm3
.....................................................................gugagcaaaguuucaggCguga................... 219 1 Fm3
.....................................................................gugagcaCaguuucaggugugа................... 7 1 Fm3
.....................................................................gugagcaaagAuucaggugugа................... 60 1 Fm3
.....................................................................gugagcaaGguuucaggugugа................... 3 1 Fm3
.....................................................................gugagcaaaguuucUggugugа................... 1 1 Fm3
.....................................................................gugagcaaaguuucagUuguga................... 36 1 Fm3
.....................................................................gugagcaaaguuucaggugCga................... 4 1 Fm3
.....................................................................gugagcaaaguuucaggAguga................... 171 1 Fm3
.....................................................................gugagcaaaguuucCggugugа................... 1 1 Fm3
.....................................................................gugagcGaaguuucaggugugа................... 5 1 Fm3
.....................................................................gugaUcaaaguuucaggugugа................... 2 1 Fm3
.....................................................................gugagcaaaguuucaggugugG................... 4 1 Fm3
.....................................................................gugagcaaagCuucaggugugа................... 40 1 Fm3
.....................................................................gugagcCaaguuucaggugugа................... 1 1 Fm3
.....................................................................gugagcaaaguuucagAuguga................... 72 1 Fm3
.....................................................................gugagGaaaguuucaggugugа................... 2 1 Fm3
.....................................................................gAgagcaaaguuucaggugugа................... 14 1 Fm3
.....................................................................gugagcaaaguuuGaggugugа................... 2 1 Fm3
.....................................................................gugagcaaaguuGcaggugugа................... 1 1 Fm3
.....................................................................gugagcaaaguuucagCuguga................... 87 1 Fm3
.....................................................................gugaAcaaaguuucaggugugа................... 3 1 Fm3
.....................................................................gugagcUaaguuucaggugugа................... 6 1 Fm3
.....................................................................gugagcaaaguuucagguguUa................... 5 1 Fm3
.....................................................................Nugagcaaaguuucaggugugа................... 14 1 Fm3
.....................................................................gugagcaaaguCucaggugugа................... 3 1 Fm3
.....................................................................Augagcaaaguuucaggugugа................... 3 1 Fm3
.....................................................................gugUgcaaaguuucaggugugа................... 5 1 Fm3
.....................................................................guCagcaaaguuucaggugugа................... 3 1 Fm3
.....................................................................gCgagcaaaguuucaggugugа................... 3 1 Fm3
.....................................................................gugagcaaaguuucagguCuga................... 1 1 Fm3
.....................................................................gugagcaaaguuucaggugugаG.................. 13 1 Fm3
.....................................................................gugagcaaaguuucaggCgugac.................. 1 1 Fm3
.....................................................................gugagcaaaguuucaggugugаU.................. 626 1 Fm3
.....................................................................gugagcaaaguuucagguCugac.................. 1 1 Fm3
.....................................................................gugagcaaUguuucaggugugac.................. 12 1 Fm3
.....................................................................gugagcaaaguuucaggugugаA.................. 1013 1 Fm3
.....................................................................gugagcaaaguuucaggugugac.................. 33 0 Fm3
.....................................................................gugagcaaaguuucaggugugаGu................. 8 1 Fm3
.....................................................................gugagcaaaguuucaggugugаAu................. 19 1 Fm3
.....................................................................gugagcaaaguuucaggugugCcu................. 1 1 Fm3
.....................................................................gugagcaaaguuucaggugugacu................. 3 0 Fm3
.....................................................................gugagcaaaguuucaggugugаUu................. 47 1 Fm3
......................................................................ugagcaaaguuucaggug...................... 1 0 Fm3
......................................................................ugagcaaaguuucaggugu..................... 1 0 Fm3
......................................................................ugagcaaaguuucaggugug.................... 4 0 Fm3
......................................................................ugagcaaaguuucagguCuga................... 7 1 Fm3
......................................................................ugagcaaaguuucaggugugа................... 918 0 Fm3
......................................................................ugagcaaaguGucaggugugа................... 5 1 Fm3
```

Star Mature

```
aaaggauuaucaaauugaugucccugucaguugugccugaaauauuugcucagaccugugguuugaaaaggugagcaaaguuucaggugugacugauagguggcgugacagc
.......................................................................ugagcaaaguuucagguAuga....................    8     1     Fm3
.......................................................................uAagcaaaguuucaggududa....................    1     1     Fm3
.......................................................................ugUgcaaaguuucaggugugа....................    1     1     Fm3
.......................................................................ugagcaaaguuucagCuguga....................    1     1     Fm3
.......................................................................ugagcaaaguuucaggAguga....................    4     1     Fm3
.......................................................................ugagcaaaguuucagguUuga....................    4     1     Fm3
.......................................................................ugagcaaaguAucaggududa....................    4     1     Fm3
.......................................................................ugagUaaaguuucaggududa....................    1     1     Fm3
.......................................................................ugCgcaaaguuucaggududa....................    1     1     Fm3
.......................................................................ugagcaaaguuucagguguqU....................    65    1     Fm3
.......................................................................ugagcaaaguuucUggududa....................    1     1     Fm3
.......................................................................uUagcaaaguuucaggududa....................    1     1     Fm3
.......................................................................ugagcaaaguuucaggugugC....................    2     1     Fm3
.......................................................................ugagcaaaguuucaggCguga....................    1     1     Fm3
.......................................................................ugagcaaaguuucGggududa....................    1     1     Fm3
.......................................................................ugagcaaaguuucaggugUAa....................    1     1     Fm3
.......................................................................ugagcaaaguuucaggugugac...................    32    0     Fm3
.......................................................................ugagcaaaguuucagguUugac...................    1     1     Fm3
.......................................................................ugagcaaUguuucaggugugac...................    15    1     Fm3
.......................................................................ugagcaaaguuucaggugugaA...................    112   1     Fm3
.......................................................................ugagcaaaguuucaggugugaU...................    97    1     Fm3
.......................................................................ugagcaaUguuucaggugugacu..................    6     1     Fm3
.......................................................................ugagcaaaguuucaggugugacu..................    6     0     Fm3
.......................................................................ugagcaaaguuucaggugugaUu..................    3     1     Fm3
.......................................................................ugagcaaaguuucaggugugaAu..................    2     1     Fm3
........................................................................gagcaaaguuucaggududa....................    1     0     Fm3
```
